# A Novel Cell-Sheet Technology That Achieves Durable Factor VIII Delivery in a Mouse Model of Hemophilia A

**DOI:** 10.1371/journal.pone.0083280

**Published:** 2013-12-16

**Authors:** Kohei Tatsumi, Mitsuhiko Sugimoto, David Lillicrap, Midori Shima, Kazuo Ohashi, Teruo Okano, Hideto Matsui

**Affiliations:** 1 Institute of Advanced Biomedical Engineering and Science, Tokyo Women's Medical University, Tokyo, Japan; 2 Department of Regulatory Medicine for Thrombosis, Nara Medical University, Kashihara, Nara, Japan; 3 Department of Pathology and Molecular Medicine, Queen's University, Kingston, Ontario, Canada; 4 Department of Pediatrics, Nara Medical University, Kashihara, Nara, Japan; National Cerebral and Cardiovascular Center, Japan

## Abstract

Gene- or cell-based therapies aimed at creating delivery systems for coagulation factor VIII (FVIII) protein have emerged as promising options for hemophilia A treatment. However, several issues remain to be addressed regarding the efficacies and adverse events of these new classes of therapies. To improve an existing cell-based therapy involving the subcutaneous transplantation of FVIII-transduced blood outgrowth endothelial cells (BOECs), we employed a novel cell-sheet technology that allows individual dispersed cells to form a thin and contiguous monolayer without traditional bioabsorbable scaffold matrices. Compared to the traditional methodology, our cell-sheet approach resulted in longer-term and 3–5-fold higher expression of FVIII (up to 11% of normal) in recipient hemophilia A mice that lacked a FVIII humoral immune response due to transient immunosuppression with cyclophosphamide. Histological studies revealed that the transplanted BOEC sheets were structured as flat clusters, supporting the long-term expression of therapeutic FVIII in plasma from an ectopic subcutaneous space. Our novel tissue-engineering approach using genetically modified BOEC sheets could aid in development of cell-based therapy that will allow safe and effective *in vivo* delivery of functional FVIII protein in patients with hemophilia A.

## Introduction

Hemophilia A is an inherited bleeding disorder caused by a deficiency of coagulation factor VIII (FVIII). Currently, patients with hemophilia A are treated with plasma-derived or recombinant FVIII concentrates [1]. This form of protein-replacement therapy has improved management of bleeding in hemophilia A patients. However, this method is also problematic because of the requirement for frequent venous access as well as the limited availability and high costs of FVIII concentrates. To address such problems, gene- or cell-based therapies are attractive alternative strategies, and such methods are now expansively being in the progress for the disease. Indeed, continuous expression of FVIII levels as low as 1–5% of normal substantially ameliorates the bleeding phenotype and improves quality of life in preclinical [2]–[5] and clinical settings [6]–[8].

We previously reported that therapeutic levels of plasma FVIII can be successfully achieved in hemophilia A mice by subcutaneous implantation of lentivirally engineered blood outgrowth endothelial cells (BOECs) mixed with Matrigel [9]. However, in that system we observed gradual loss of plasma FVIII, probably due to breakdown of the scaffold material or cell death.

To overcome these issues, we employed cell-sheet technology, an innovative tissue-engineering approach that allows individual dispersed cells to form a thin and contiguous monolayer; this method has recently shown great promise in regenerative medicine [10]–[11]. In fact, our previous studies [12]–[13] indicated that cell sheets engineered from a number of sources have considerable benefits, and can strengthen the viability and functionality of cells implanted in the subcutaneous space for therapeutic purposes. Here, we report a unique and effective tissue-engineering approach using BOEC sheets as a new class of potential cell-based treatment for hemophilia A.

## Materials and Methods

### Animals

Immunocompetent C57Bl/6 hemophilia A mice with targeted destruction of exon 16 of the *FVIII* gene [14] were a kind gift from Prof. Yoichi Sakata (Jichi Medical University, Shimotsuke, Japan). Wild-type C57Bl/6 mice syngenic to the hemophilia A mice were used as donors of normal mouse plasma. All animal procedures were reviewed and approved by the Animal Care Committee at Nara Medical University.

### Isolation and lentiviral vector transduction of BOECs *in vitro*

Isolation of BOECs from hemophilia A mice and *in vitro* FVIII transduction of hemophilia A mouse BOECs, using a lentiviral vector that encodes the canine B-domain deleted FVIII (BDD-FVIII) under the control of the EF1-alpha (EF1α) promoter, were described previously [9], [15]. In brief, cultured murine BOECs (1×10^5^) were transduced following single exposure of the Lenti- EF1α-cFVIII viral vectors at increasing multiplicities of infection (MOI). After transduction, cells were expanded, and assessment of FVIII expression from BOECs was carried out using a functional chromogenic assay described below.

### Fabrication of genetically modified BOEC sheets

The lentivirally modified hemophilia A mouse BOECs expressing canine FVIII were seeded on temperature-responsive culture dishes (UpCell, CellSeed, Tokyo, Japan) [10]–[11]. The dishes were created by covalently grafting Poly (N-isopropylacrylamide) (PIPAAm) by electron-beam irradiation. Normal- and large-sized cell sheets were generated using 35-mm and 100-mm dishes, respectively. When cultured BOECs reached confluency, they were detached from PIPPAm dishes as uniformly connected tissue sheets by lowering the culture temperature to 20°C for 30 min.

### Transplantation of BOEC sheets to hemophilia A mice

Cell counting revealed that normal-sized and large-sized BOEC sheets consisted of 2.8±0.4×10^5^ and 2.0±0.2×10^6^ cells, respectively. BOEC sheets were recovered with support membranes for transplantation into subcutaneous sites in hemophilia A mice. To avoid excessive surgical procedure–related bleeding, all recipient hemophilia A mice received an intraperitoneal injection of 0.5 mL pooled normal mouse plasma 30 min prior to surgical procedures. All surgeries were conducted under general anesthesia using isoflurane. Because canine FVIII is inherently immunogenic in hemophilia A mice, some recipient mice also received intraperitoneal injection of cyclophosphamide (20 mg/kg per injection) administered on the day of transplantation and then biweekly for 4 weeks. All recipient hemophilia A mice that did not receive this treatment developed an anti–canine FVIII humoral immune response.

### FVIII activity, FVIII antigen and FVIII antibody assays

Functional FVIII was quantified by a chromogenic assay as previously described [9]. FVIII antigen was calculated by canine FVIII ELISA kit (Affinity Biologicals, Ancaster, ON, Canada). Development of anti–canine FVIII humoral response was detected and quantitated by the Bethesda assay [16]. The standard curve was generated with pooled normal canine plasma. Same mouse plasma samples were used in these assays.

### Tail-clip bleeding tests

Successful long-term phenotypic correction was tested in both untreated wild-type mice and hemophilia A mice that received transplants of BOEC sheets. At the termination of the experiments, phenotypes were analyzed by anesthetizing the mice with isoflurane and clipping the tails at the position where the tail diameter was 0.5 mm. The mice were then observed for 1 hour to determine the bleeding time.

### Subcutaneous implant removal and immunohistochemical analysis

Eight weeks after transplantation, some mice were sacrificed. The implants of sacrificed mice were recovered, fixed with 4% formalin, embedded in paraffin, and sectioned for hematoxylin and eosin (H&E) staining. To assess FVIII expression in BOECs, specimens were characterized with double immunostaining for FVIII and vWF as previously described [9]. Specimens were viewed with a confocal laser scanning microscope (CLSM, FV300; Olympus Co., Tokyo, Japan). H&E and immunostaining were performed on sequential sections.

## Results and Discussion

Recent preclinical and clinical studies using adeno-associated viral (AAV) vectors for hemophilia B demonstrated that the safety profile is partly determined by vector dose, and that immune responses to AAV-capsid proteins with subsequent hepatocyte toxicity require transient immunosuppression in order to achieve sustained transgene expression [17]–[20]. However, some concerns still remain regarding the safety of systemic injection of viral vectors. Potential side effects include adverse immunological reactions, vector-mediated cytotoxicity, germ-line transmission, and insertional oncogenesis [21]–[23]. Moreover, especially in hemophilia A, an alternative transgene delivery approach may be necessary due to the large size of the FVIII cDNA. Therefore, considering the aforementioned issues, we elected to investigate an *ex vivo* gene-transfer strategy that avoids systemic administration of a viral vector.

In the Transkaryotic Therapy study, the first *ex vivo* gene-transfer strategy for hemophilia A patients in the clinic, the limited viability of the implanted autologous fibroblasts failed to provide sustained therapeutic levels of FVIII [8]. In this regard, tissue-engineering approaches using cell-sheet technology have already been applied in different clinical settings as therapeutic modalities for several diseases, including corneal disease [24], wounds of the esophageal mucosa [25], heart failure [26], and periodontitis [27]. In addition, we recently demonstrated that cell-sheet transplantation using pancreatic islet cells can successfully improve disease in a mouse model of diabetes mellitus [13]. Thus, cell-sheet technology represents a new class of drug-delivery system, allowing engineering of tissues that can secrete therapeutical proteins such as insulin.

**Figure 1:**
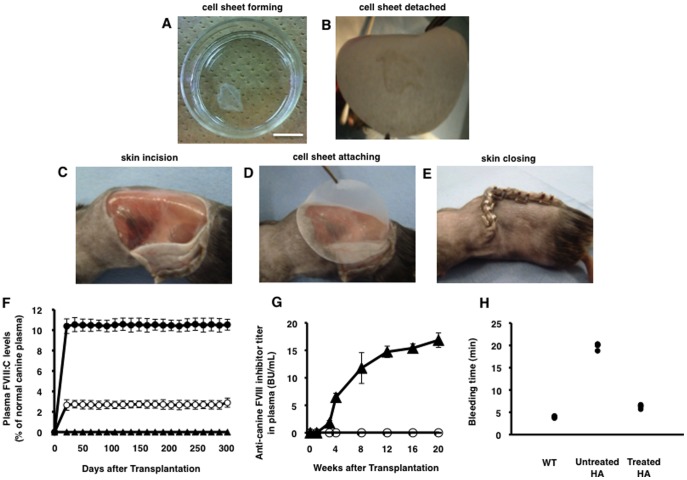
Subcutaneous transplantation of canine FVIII (cFVIII)-transduced blood outgrowth endothelial cell (BOEC) sheets in hemophilia A mice. (A–E) Schematic procedure for BOEC sheet transplantation. BOECs from hemophilia A mice were transduced using a lentiviral vector expressing the canine FVIII gene. The cells were cultured on temperature-responsive culture dishes. (A) Cell sheets were detached from the culture dishes by lowering the culture temperature, and (B) harvested as monolayer sheets using a support membrane. The scale bar represents 10 mm. (C) L-shaped skin incisions were made on the left dorsal regions of hemophilia A mice. (D) BOEC sheets were transplanted into the sites. After 5 min of attachment, the support membrane was carefully removed. (E) Thereafter, the skin flap was returned to its original position, and the skin wound was closed. (F) Plasma FVIII activity (FVIII: C) levels after cFVIII-transduced BOEC sheet transplantation in hemophilia A mice. Original-size sheets (open circles, n = 7) and large-size sheets (filled circles, n = 5) were fabricated on 35-mm and 100-mm-sized culture dishes, respectively. BOEC sheets not subjected to gene transduction were also transplanted (filled triangles, n = 2). (G) Anti-cFVIII inhibitor titers after transplantation of cFVIII-transduced (filled triangles, n = 4) or non-transduced (open circles, n = 3) BOEC sheets in hemophilia A mice that did not receive cyclophosphamide. (H) Bleeding time after tail clipping in wild-type mice (n = 4), hemophilia A mice (n = 5), and hemophilia A mice that were treated with large-sized cFVIII-expressing BOEC sheets and cyclophosphamide administration (n = 5).

In this context, we employed endothelial cells formed into a contiguous monolayer sheet, which can be readily transplanted into the subcutaneous space for the production of FVIII (Figure 1A–1E). Under transient immunosuppression with cyclophosphamide, plasma FVIII levels up to 11% of normal were detected 3 weeks after transplantation in immunocompetent hemophilia A mice receiving transplantation of BOEC sheets. These levels were sustained for at least 300 days of observation without the development of anti-FVIII antibodies (Figure 1F–G). In addition, the levels of canine FVIII antigen by canine FVIII-specific ELISA are corresponded with canine FVIII activity by chromogenic assay in same plasma samples (data not shown). The levels and duration of FVIII expression achieved using this method were much higher than those observed in our previous BOEC studies [9], in which cell-sheet technology was not used. In the earlier study, elevated FVIII in plasma (maximum activity: 2% of normal) fell to zero 180 days after transplantation of BOECs. Consistent with increased FVIII activity, tail-clipping tests revealed that bleeding was significantly shortened in hemophilia A mice that received BOEC sheet transplants (Figure 1H). Together, these results clearly demonstrate that long-term phenotypic correction of hemophilia A in this mouse model was successfully achieved using endothelial cells in conjunction with a novel cell-sheet technology.

**Figure 2:**
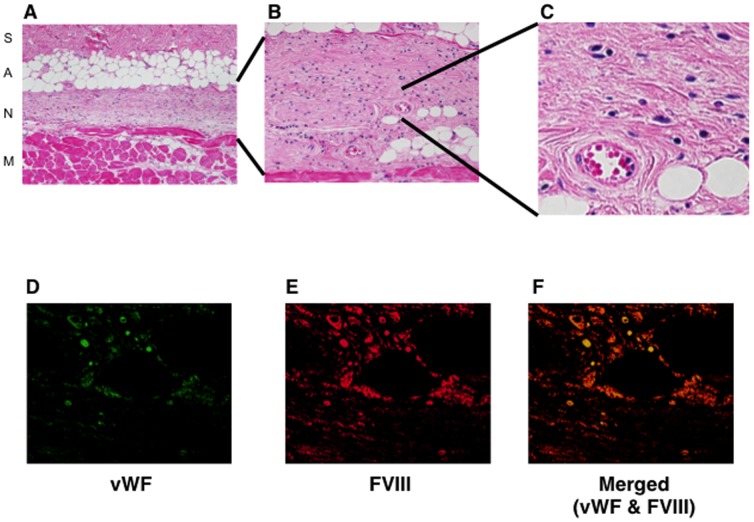
Histological analyses of subcutaneously transplanted genetically modified blood outgrowth endothelial cell (BOEC) sheets in hemophilia A mice. Eight weeks after transplantation of canine FVIII-transduced BOEC sheets, several recipient hemophilia A mice were sacrificed, and the implant tissue sections were subjected to (A–C) hematoxylin and eosin staining and immunostaining for (D) von Willebrand Factor (vWF) or (E) FVIII. (F) Merged image of vWF and FVIII staining. Magnification: (A) ×10, (B) ×20, (C) ×40, (D–F) ×60. S, skin; A, adipose tissue; N, newly generated tissues including BOEC sheet transplants and connective tissues; M, muscle. Each scale bar represents 30 µm. Engrafted BOEC implants were structured as flat sheets without any cell infiltration. Moreover, FVIII and vWF double-positive vessels were observed in newly generated tissues derived from the implanted BOECs. Abbreviations: H&E, hematoxylin and eosin; vWF, von Willebrand factor; FVIII, factor VIII.

Histological observations confirmed the superior outcome of our novel cell-sheet approach. In particular, histological studies revealed clear tube formation by FVIII-positive BOECs in the sub-adipose tissue layer, suggesting that the transplanted BOECs could integrate efficiently into the subcutaneous space and differentiate into mature endothelial cells, leading to formation of new blood vessels without any cellular response (Figure 2A–2F). Furthermore, these histological observations verified that cell viability was much improved in the novel cell-sheet approach, resulting in longer-term and 3–5-fold higher expression of plasma FVIII per numbers of transplanted BOECs, relative to our previous Matrigel transplantation approach [9].

The use of a temperature-responsive poly (N-isopropylacrylamide) (PIPAAm)-grafted dish may also explain the superior outcome of our novel BOEC sheet approach. Such dishes allow simple detachment of cultured cells without the use of proteolytic enzymes such as trypsin and the efficient harvest of a cell sheet as a contiguous monolayer that retains its native intercellular communications and intracellular microstructure. These properties of PIPAAm-grafted dishes could contribute to the preservation of normal cellular functions. In addition, BOECs in monolayer sheet configuration may facilitate oxygen delivery within the tissue microenvironment. In our previous study, in which BOECs were transplanted with Matrigel [9], the generation of BOEC clusters might not have provided adequate perfusion of the cells with nutrients, because the subcutaneous space was not as actively vascularized. By contrast, our novel cell-sheet approach allows unlimited diffusion of gases required for cell survival, thereby contributing to improved cell viability. Several previous studies have been designed around the development of vascular platforms within the subcutaneous space in hopes of enhancing cell survival [28]. In this regard, it is noteworthy that our novel approach does not require the preparation of a vascular platform before cell transplantation.

Compared to recently developed gene therapies that employ systemic administration of viral vectors, our novel BOEC sheet approach has considerable benefits. Indeed, this cell-sheet transplantation approach can be repeated several times in a single recipient, if necessary, in order to increase the therapeutic efficacy.

In order to advance our mouse study into the clinic, there remain several issues to be addressed. Perhaps most importantly, the size of cell sheets used for transplantation must be significantly enlarged for use in human hemophiliacs. Development of multilayer cell-sheet transplantation within a confined space may provide a solution to this problem, and research on this topic is now underway in our laboratory.

## Conclusion

We have succeeded in long-term phenotypic correction of hemophilia A in a mouse model by *ex vivo* engineering of genetically modified endothelial cells in an ectopic subcutaneous space. Our novel approach using cell sheet technology could represent an initial basis for curative treatment of hemophiliacs in the near future.
